# Comparison of Artificial Neural Networks and Response Surface Methodology towards an Efficient Ultrasound-Assisted Extraction of Chlorogenic Acid from *Lonicera japonica*

**DOI:** 10.3390/molecules24122304

**Published:** 2019-06-21

**Authors:** Hui-Chuan Yu, Shang-Ming Huang, Wei-Min Lin, Chia-Hung Kuo, Chwen-Jen Shieh

**Affiliations:** 1 Biotechnology Center, National Chung Hsing University, 250 Kuokuang Road, Taichung 40227, Taiwan; cathy_yu55@hotmail.com (H.-C.Y.); zxzxmj2323@hotmail.com (S.-M.H.); 2 Department of Chemical Engineering, National Chung Hsing University, 145 Xingda Road, Taichung 40227, Taiwan; lin78115@hotmail.com; 3 Department of Seafood Science, National Kaohsiung University of Science and Technology, 142 Haijhuan Road, Nanzih District, Kaohsiung 811, Taiwan

**Keywords:** *Lonicera japonica*, chlorogenic acid, extraction, optimization, response surface methodology, artificial neural networks

## Abstract

Chlorogenic acid (CGA), a bioactive compound commonly found in plants, has been demonstrated possessing nutraceutical potential in recent years. However, the more critical issue concerning how to improve production efficacy of CGA is still limited. It is a challenge to harvest a large amount of CGA without prolonging extraction time. In this study, the feasibility of using ultrasound for CGA extraction from *Lonicera japonica* was investigated. A central composite design (CCD) was employed to evaluate the effects of the operation parameters, including temperature, ethanol concentration, liquid to solid ratio, and ultrasound power on CGA yields. Meanwhile, the process of ultrasound-assisted extraction was optimized through modeling response surface methodology (RSM) and artificial neural network (ANN). The data indicated that CGA was efficiently extracted from the flower of *Lonicera japonica* by ultrasound assistance. The optimal conditions for the maximum extraction of CGA were as follows: The temperature at 33.56 °C, ethanol concentration at 65.88%, L/S ratio at 46:1 mL/g and ultrasound power at 150 W. ANN possessed greater optimization capacity than RSM for fitting experimental data and predicting the extraction process to obtain a maximum CGA yield. In conclusion, the process of ultrasound-assisted extraction can be well established by a methodological approach using either RSM or ANN, but it is worth mentioning that the ANN model used here showed the superiority over RSM for predicting and optimizing.

## 1. Introduction

Phenolic acids have received a great deal of attention over the years due to its beneficial biological activities. CGA is a common phenolic acid abundantly found in the coffee-related natural products, vegetables, and fruits [1]. Structurally, CGA is a family of esters formed between caffeic acid and quinic acids. Neochlorogenic acid (5-*O*-caffeoylquinic acid) is a very common isomer in CGA. It has been widely demonstrated that CGA shows diverse bioactivities and health-promoting functionality such as hepatoprotective, cardioprotective, anti-inflammation, antioxidant, anti-cancer, and anti-bacteria [2]. However, literature has indicated that the high-temperature water extraction process can lead to CGA isomerization, transformation and degradation [3], consequently reducing the antioxidant activity [4,5] and other biological activity [3] of CGA.

The conventional extraction method of CGA, e.g., the Soxhlet extraction method or flask-shaking method is time-consuming, generally exhibits low production efficiency [5] and low reproducibility [3]. Moreover, the extraction process requires large amounts of solvent and continuous heating at a high temperature. In order to improve the yield and the quality of final products, the use of ultrasound for bioactive compound extraction is a feasible approach to achieve a reduction in extraction time and solvent consumption [3,6].

Ultrasound causes cavitation by alternating low-pressure and high-pressure waves in liquids [7]. As the cavitation bubbles collapse, the energy released, leading to an intense shock wave and largely turbulent flow in the liquids. These turbulent flow further produce strong shear forces and damage to plant materials [7]. These mechanical effects of ultrasound have four significant benefits for extraction of plant substance, (a) assisting the release of contents after breaking the cell walls, (b) promoting more penetration of solvent into the interior of the cell and enhancing the mass transfer [8], (c) reducing extraction time at lower processing temperatures [9] and (d) reducing the degradation of products extracted from plants [3]. For ultrasound-mediated plant extraction, the operating parameters such as irradiation time and sonication power were believed to have dependent effects on extraction yield [10,11]. Li et al. [10] reported an ultrasound-assisted extraction of CGA from fresh leaves of *Eucommia ulmodies Oliv*. They found that the sonication method exhibited highly efficient in the extraction of CGA from *E. ulmodies* compared with traditional methods. The optimum extraction conditions were found to be aqueous methanol of 70%, solvent/sample ratio of 20:1 (*v*/*w*) and extraction time of 30 min with three cycles. Moreover, Goltz et al. [12] investigated the extraction process of the phenolic compounds from macela by ultrasonic assistance. They found that ultrasound-assisted extraction not only increased the yield but also improved the antioxidant activity of the extracts. Mazvimba et al. [13] studied the application of heat reflux and ultrasonic-assisted extraction techniques for the extraction of CGA from dry tobacco leaves. Although the heat reflux extraction method with methanol showed high extraction efficiency, the existence of methanol could cause the adulteration of extracts. Ultrasound-assisted aqueous extraction process enhances CGA solubility in water, reduces 25% of solvent consumption, shortens the extraction time from 3.5 h per cycle to only 15 min per cycle and improves extraction efficiency from dried tobacco leaves.

Response surface methodology (RSM) has been widely applied to optimize the process of bioactive component extraction, which evaluates the relative importance of each independent variable and determines the optimal operating conditions for the predicted responses. It has been successfully applied to optimize parameters in various chemical processes [14,15,16,17]. However, any form of a non-linear relationship between the variables may result in a decrease in the prediction accuracy of the RSM [17]. Recently, an artificial neural network (ANN) has been developed as an alternative to the RSM system for complex non-linear multivariate modeling. ANN performs the project by learning from training examples and does not need any prior knowledge of the correlation between targeted responses [17,18]. As compared to the RSM, ANN could be a powerful tool to propose higher accuracy and efficiency on the fitting of experimental responses, prediction, and modeling of biochemical processes [16,17,18]. Presently, the information about the production of CGA from *Lonicera japonica* in the ANN system is still limited [13,19]. 

In this study, the efficiency of CGA extraction was determined by ultrasonic-assisted extraction. On the other hand, RSM and ANN methods were conducted for modeling and optimizing the process of ultrasonic-assisted extraction. The process was performed using a 5-level-4-factor central composite rotatable design (CCRD) to develop RSM and ANN models. Results were statistically compared by the coefficient of correlation determination (*R*^2^), root mean square error (RMSE), and absolute average deviation (AAD).

## 2. Results and Discussion

### 2.1. Single-Factor Experiments

**Figure 1:**
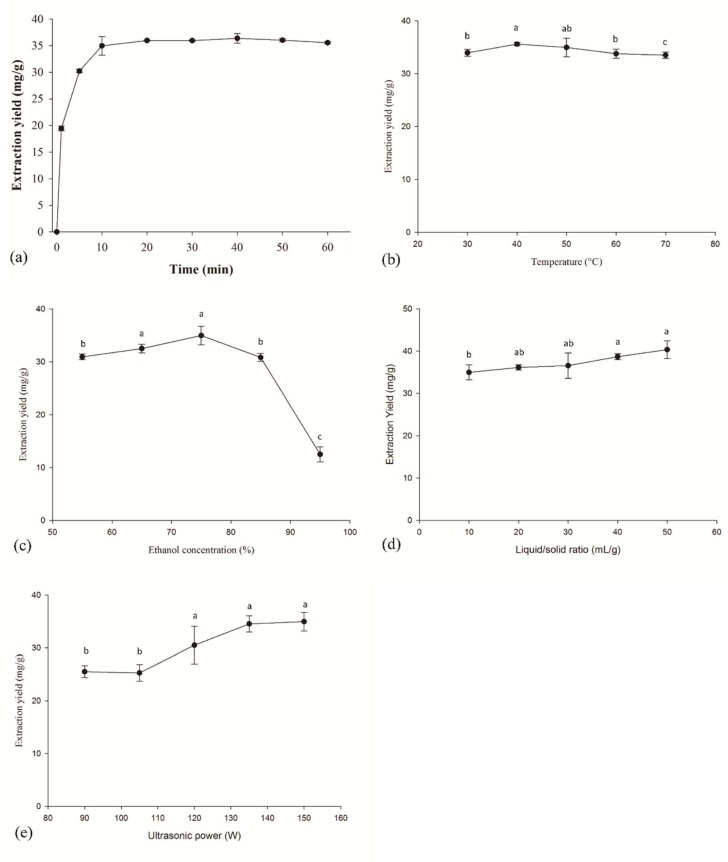
Effect of (**a**) time, (**b**) temperature, (**c**) ethanol concentration, (**d**) liquid/solid ratio, and (**e**) ultrasonic power on the yield of CGA. Different letters a, b, and c indicate significant differences (*p* < 0.05).

As shown in Figure 1a, the extraction yield increased as the extraction time increased under the experimental conditions of indicated L/S ratio (10 mL/g), a reaction temperature of 50 °C, ultrasonic power of 150 W, and ethanol concentration of 75%. When the extraction time was in the range from 0 to 10 min, the yield of CGA tended to rise rapidly and reached a maximum value (35 mg/g). When the extraction time continued to increase, the yield of CGA nearly remained constant. Considering the extraction yield and production cost of the process, the extraction time of 10 min was selected for CGA extraction from *Lonicera japonica* in a further experiment.

The effect of extraction temperature on the yield of CGA under the experimental conditions of L/S ratio (10 mL/g), an extraction time of 10 min, ultrasonic power of 150 W, and ethanol concentration of 75%, was shown in Figure 1b. The result indicated that the extraction yield of CGA did not significantly vary at different extraction temperatures from 30 °C to 70 °C. Mazvimba et al. [13] reported that the maximum extraction yield of CGA from tobacco leaves was achieved under 70 °C of extraction temperature, 3.5 h of extraction cycle time, and 80% of methanol. Hu et al. [19] indicated that some chemical structures of phenolic acids might change at a higher temperature, leading to a loss of biological activity. Therefore, performing an extraction at lower temperatures can improve functional properties and reduce energy consumption.

The effects of ethanol concentration on the yield of CGA under the experimental conditions of L/S ratio (10 mL/g), an extraction time of 10 min, ultrasonic power of 150 W, and a reaction temperature of 50 °C, was shown in Figure 1c. The extraction yields firstly increased, and then decreased. The maximum yield was observed in 34.96 mg/g with the treatment of 75% ethanol concentration. Hu et al. [19] indicated that the use of ethanol at high concentrations might change the solvent polarity, and then the solubility of CGA was affected. Li et al. [10] suggested that 70% of methanol and 90 min of extraction time could recover the most CGA from leaves of *Eucommia ulmodies Oliv*.

As shown in Figure 1d, the yield of CGA was increased as the L/S ratio increased. This result can be explained by mass transfer. When a higher solvent to solid ratio was used, the diffusion rate increased, leading to an increase in the extraction yield. However, not only solvent to solid ratio but also temperature and composition of the solution affected the extraction yield of the total phenolic compound [20]. Water/ethanol mixtures as extractants can reduce the generation of free radical from the decomposition of water because ethanol is more stable in terms of homolytic cleavage [3]. Cacace and Mazza [20] studied the optimization efficiency on the extraction of anthocyanins and other phenolic compounds from black currants by using aqueous ethanol. The results showed that the solvent to solid ratio has a critical role in the extraction efficiency of phenolic content. Phenolic content increased as ethanol concentration increased (up to 60%), and then phenolic content decreased as ethanol concentration further increased. As shown in Figure 1e, the yield of CGA was increased as ultrasonic power increased. The maximum yield was observed at 150 W of ultrasonic power. Higher power ultrasound exhibited more significant effect on cell damage and therefore improved the extraction efficiency [11].

### 2.2. RSM Model

Temperature, ethanol concentration, L/S ratio, and ultrasonic power are the crucial parameters affecting the extraction efficiency of CGA. In this study, the ranges of optimal temperature, ethanol concentration, L/S ratio, and ultrasonic power were 30C–70 °C, 55–95%, 10–50 mL/g, and 90–150 W, respectively (Table 1 and Table 2). The yields of CGA were obtained between 13.18 mg/g and 41.64 mg/g. The second-order response model obtained was as follows:

*Y*(mg⁄g) = −344.708589+2.212185*X*_1_+5.044251*X*_2_+3.887346*X*_3_+1.337813*X*_4_+0.008599*X*_1_*X*_2_

−0.017324*X*_1_*X*_3_−0.011583*X*_1_*X*_4_−0.012547*X*_2_*X*_3_−0.000117*X*_2_*X*_4_−0.006649*X*_3_*X*_4_ (1)

−0.007313*X*_1_^2^−0.03598*X*_2_^2^−0.015413*X*_3_^2^−0.002116*X*_4_^2^

As shown in Table 3, the linear term of ethanol concentration (*X*_2_) and L/S ratio (*X*_3_) and the square term of ethanol concentration (*X*_2_^2^) had significant (*p* < 0.05) influences on the yield of CGA. The variance analysis of the model showed that the *p* value for the model was 0.0238, indicating that the model was significant and could monitor the optimization [21]. However, the *R*^2^ value was 0.7913, indicating that the model only explained 79.13% of the variation in the data. Moreover, the lack-of-fit (*p* < 0.05) was significant, suggesting that the regression model was inadequate to describe the observed data variations. Therefore, ANN was used to improve and obtain a more precise prediction.

### 2.3. ANN Model

**Figure 2:**
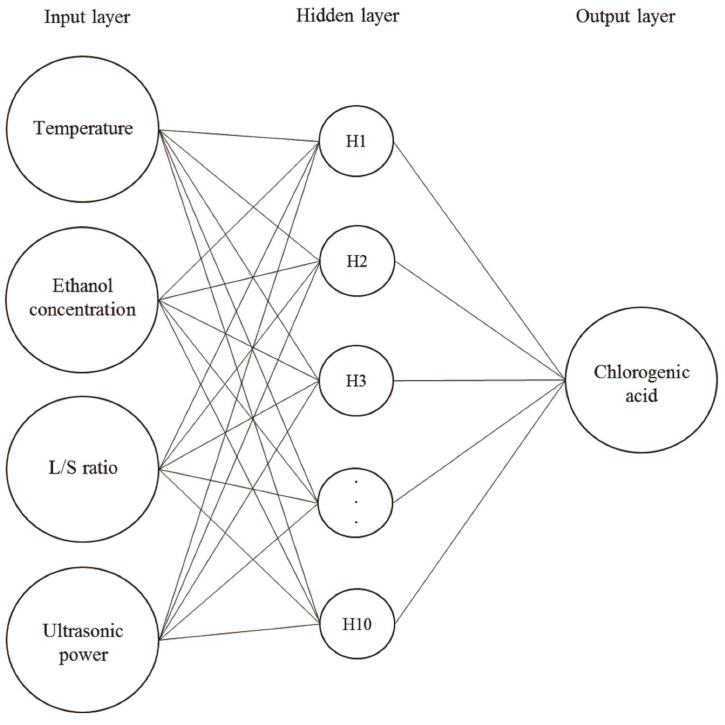
Neural network topology. The topology of multilayer feed forward neural network for the estimation of ultrasound-assisted extraction of chlorogenic acid (CGA).

**Figure 3:**
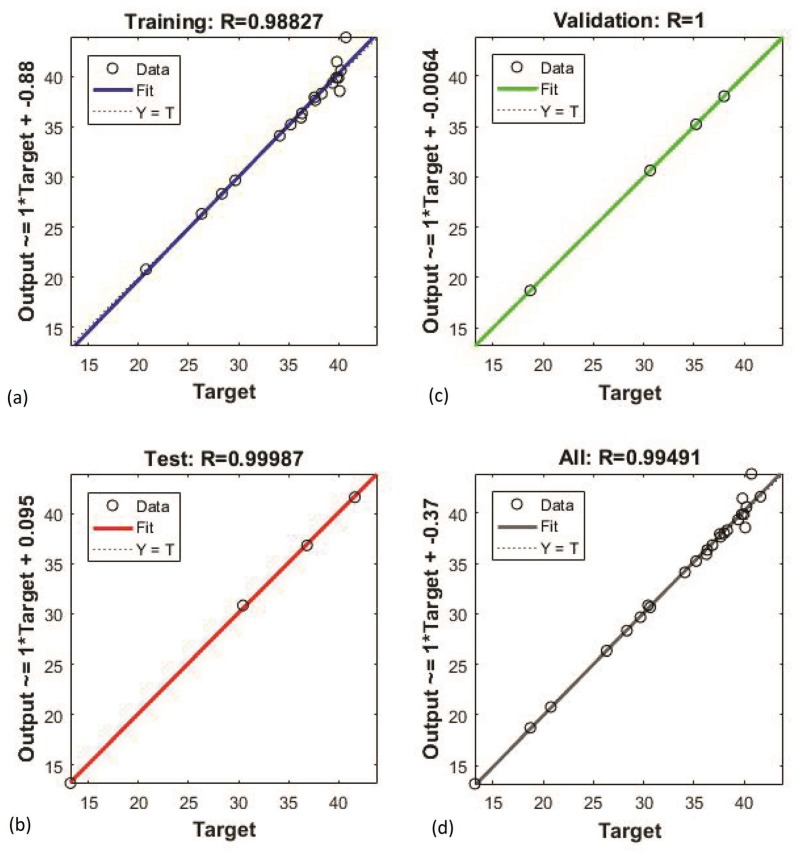
Scatter plot between experimental and predicted yield by artificial neural network (ANN) for (**a**) training, (**b**) testing, (**c**) validation, and (**d**) overall data fitting.

ANN was an effective tool for modeling unknown or semi-unknown processes. It has been applied in modeling to control the nonlinear multivariate process [22]. ANN can improve prediction accuracy to optimize the process condition of extraction from *Lonicera japonica*. In this study, 70% of the CCRD data (Table 2) were used to train the neural network model, 15% of the CCRD data were used to test, and 15% of the CCRD data were used to verify. The experimental data was divided into three parts in order to measure the performance of the neural network and predict the unobserved data [23]. Various learning algorithms were tested for training neural network models, and the best ANN model with a 4-10-1 topology was finally established (Figure 2). Figure 3 indicated that the neural network between the experimental and predicted data for training, testing, and validation fitted well. To conclude, the new construction ANN model can predict the yield of CGA.

**Figure 4:**
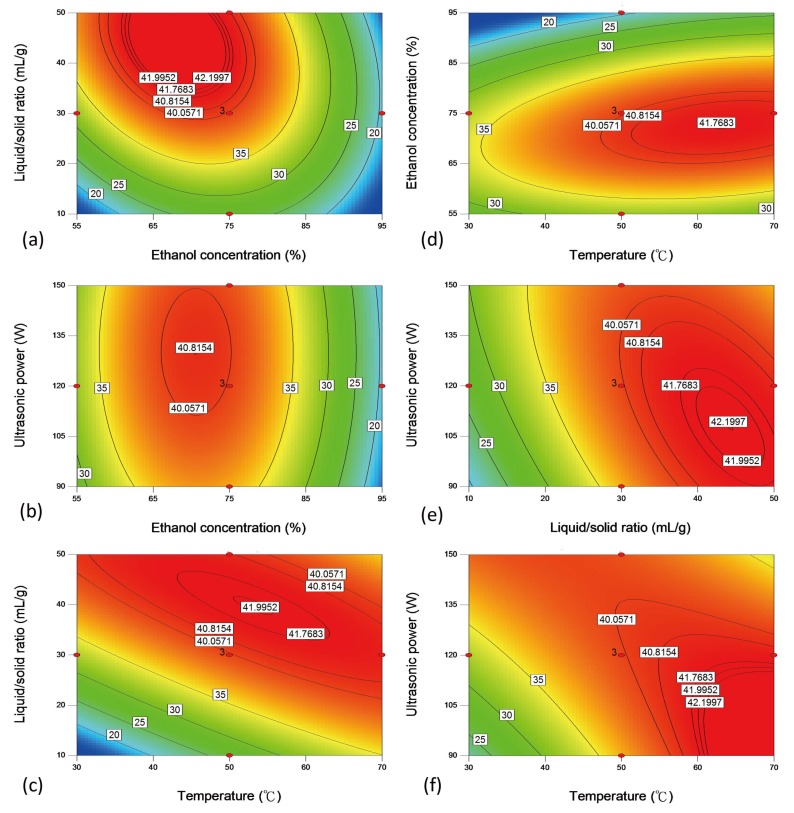
Contour plots are showing the relationships between responses variable and independent variables. (**a**) Ethanol concentration compared to L/S ratio; (**b**) ultrasonic power compared to ethanol concentration; (**c**) L/S ratio compared to temperature; (**d**) ethanol concentration compared to temperature; (**e**) ultrasonic power compared to L/S ratio; (**f**) ultrasonic power compared to temperature.

Table 2 indicates that the extraction yield predicted by ANN were superior to those predicted by RSM. The ANN-predicted values were very close to the actual yields. However, there was some difference between the predicted values of RSM and the actual yields (Table 2). As shown in Figure 4, the effect of each pair of independent variables on the yields of CGA was shown using contour plots. Figure 4a indicates that the maximum yield was found when the ethanol concentration was 65–75% and that the L/S ratio was 30–50 mL/g. The changes in extraction yield are related to the polarity of the solvent [19]. Figure 4b illustrates the effects of ethanol concentration and ultrasound power on the extraction yield, indicating that the highest yield was obtained by treatment with ethanol concentration between 65% and 75%, but ultrasound power had no effect on extraction yield. Figure 4c illustrates the effects of L/S ratio and temperature on extraction yield, indicating that the maximum yield was obtained at L/S ratio of 30–50 mL/g, while the temperature increasing from 40–60 °C did not increase the yield. As the liquid to solid ratio increased, the effect of temperature on yield decreased. Figure 4d illustrates that the maximum yield was observed at an ethanol concentration range of 65–75% and a temperature range of 50–70 °C, while there was no increase in yield over this temperature range. The contour plot showed that an extraction temperature above 70 °C might have similar extraction yield to a temperature range of 50–70 °C, however, the higher extraction temperature may cause CGA to be affected by temperature. Wianowska and Gil [3] indicated that CGA was prone to intramolecular isomerization, transesterification, and degradation at high temperatures. On the other hand, the results of the single factor experiment (Figure 1b) showed that the yield of CGA gradually decreased above 40 °C. Therefore, the experimental design did not perform a higher extraction temperature to investigate the extraction yield. Figure 4e–f illustrates that the highest yield was observed at an L/S ratio between 30 mL/g and 50 mL/g, while the temperature increasing from 50 to 70 °C did not increase the yield. It can be found that except for the ultrasonic power, the extraction temperature, the liquid-solid ratio, and the ethanol concentration are important parameters affecting the yield. At a fixed temperature (50 °C), it can observe that the ultrasonic effect on CGA extraction is significant (Figure 1e). At lower temperature (30–50 °C), the extraction yields increased with increasing ultrasonic power. The increase of ultrasonic power led to more rapid speed to generate cavitation. Thus, the extraction process was enhanced [24]. However, the increased temperature may accelerate the formation of free radicals during the ultrasonic process [25]. Thus, the total phenolic content decreased [25]. This phenomenon may explain why ultrasonic power has a less significant effect on yield in RSM modeling (Figure 4f). A contour plot of the experimental model described the relationship between the two factors and the response; an elliptical contour plot indicated the interactions between the factors were significant. The red area of the contour plot showed the range of high extraction yields, and extraction temperatures were interpreted in the ranges of 30–70 °C (Figure 4c,d,f). However, the maximum response operating condition based on the interaction between the four factors. Therefore, the most appropriate extraction temperature was not in the range of 50–70 °C.

### 2.4. Verification, Comparison and Optimization

**Figure 5:**
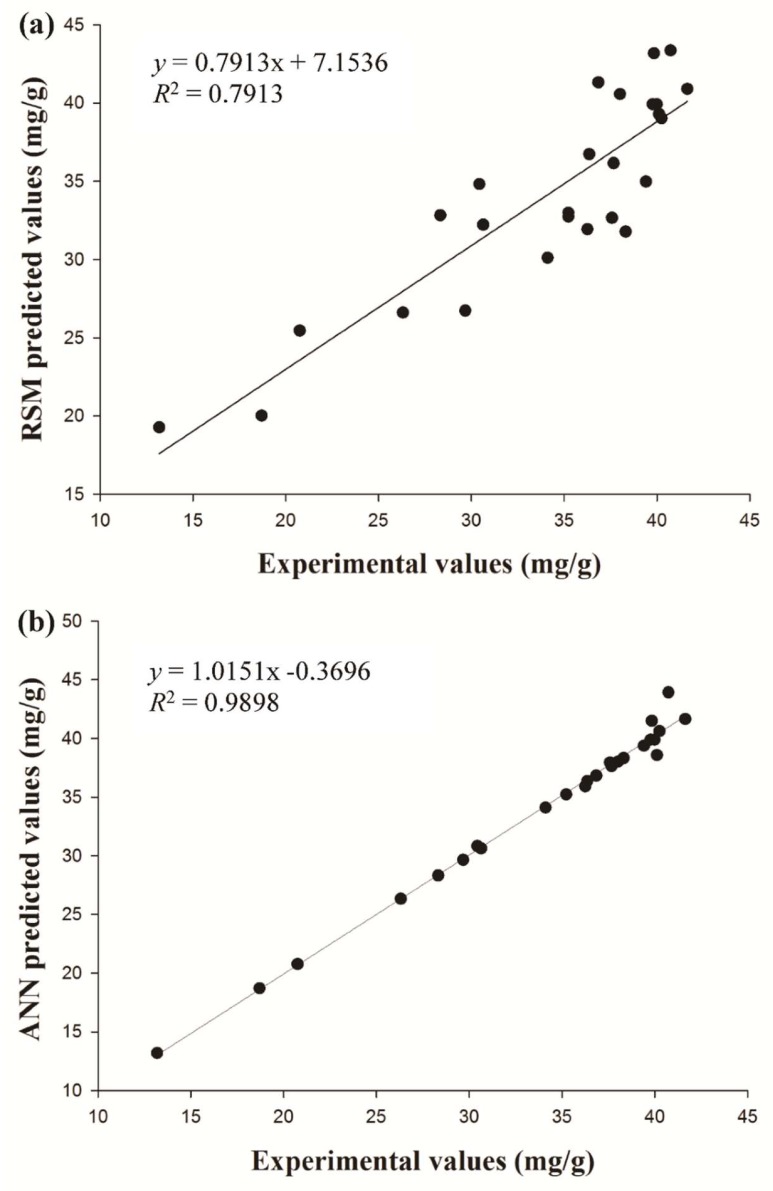
Comparison of experimental data with the predicted value obtained by (**a**) the RSM and (**b**) ANN models.

In order to verify the predictive capability of both the ANN and RSM models, a new set of experimental condition combinations was performed and did not part of the training data set (Table 4). Table 5 shows the statistical comparison of the RSM and ANN models. *R*^2^ values for constructed RSM and ANN were exhibited as 0.7913 and 0.9898 (Figure 5). It indicated that the ANN model has better performance for prediction than RSM. The RMSE for RSM and ANN was found as 1.9050 and 0.7006. The AAD for RSM and ANN was found as 1.6541 and 0.4204. Compared with the ANN model, RSM had a higher prediction error. From the results, the prediction of the ANN model has higher prediction accuracy in approximating the actual experimental values.

The following optimal conditions for ultrasound-assisted extraction for CGA from *Lonicera japonica* were determined using a ridge max analysis of an experimental model: Temperature (*X*_1_), 33.56 °C; ethanol concentration (*X*_2_), 65.88%; L/S ratio (*X*_3_), 46 mL/g; and ultrasonic power (*X*_4_), 150 W; the maximum yield was predicted to be 45.63 mg/g and 44.78 mg/g from the RSM and ANN model, respectively. Under these conditions, the actual experimental yield was 43.13 mg/g. The results confirmed that the constructed model adequately predicted the extraction yield of CGA. However, it has been proven again that the prediction ability of ANN was superior to RSM, and an optimum condition of extraction can be obtained more accurately. Compared to literature, Xu et al. [24] indicated that 37.07 mg/g of CGA was obtained by ultrasound-assisted extraction using a response surface methodology. Lin et al. [15] developed the enzyme-assisted ultrasonic extraction of resveratrol from *P. cuspidatum*, suggesting that the ANN model demonstrated more accurately in data fitting as compared to the RSM model. The results are consistent with this study. The present study demonstrated that under multi-variable-multi-level operating conditions, ANN modeling could replace RSM modeling as an operational tool for optimizing processes.

## 3. Materials and Methods 

### 3.1. Materials

*Lonicera japonica powder* was purchased from He-Kang Chinese Medicine Co. (New Taipei City, Taiwan). CGA was purchased from Acros Organics (Pittsburgh, PA, USA). Ethanol was purchased from Taiwan Tobacco & Liquor Corporation (Taipei, Taiwan). Methanol was purchased from Aencore Chemical Co. (New South Wales, Australia). Acetic acid was purchased from Sigma-Aldrich Co. (St. Louis, MO, USA). All other chemicals and reagents were analytic grade.

### 3.2. Conventional Shaking Extraction of CGA from Lonicera japonica

The extracts were prepared according to the method of Chen et al. [14] and Hsu et al. [26]. *Lonicera japonica* flowers powder (0.1 g) was mixed with 1 mL of 75% EtOH in a cap-sealed glass tube. The mixture was extracted at 50 °C for various times in an orbital shaking bath (100 rpm). After extraction, the supernatant of the sample was collected by centrifugation (C2400-P, Labnet International, Inc., Cary, NC, USA) at 13,000 rpm for 10 min. The supernatants were filtered through a 0.45 μm PVDF syringe filter and stored in a dark room at 4 °C until analysis.

### 3.3. Ultrasonic-Assisted Extraction of CGA from Lonicera japonica

*Lonicera japonica* flowers powder (0.1 g) was mixed with 1 mL of 75% EtOH in a cap-sealed glass tube. The mixture was extracted at 50 °C and 150 W of ultrasonic power with various times in an ultrasonic bath (40 kHz, Delta DC150H, Dogger Science, New Taipei, Taiwan). After extraction, the supernatant of the sample was collected by centrifugation at 13,000 rpm for 10 min. The supernatants were filtered through a 0.45 μm PVDF syringe filter and stored in a dark room at 4 °C until analysis.

### 3.4. HPLC Analysis of Products Extracted from Lonicera japonica

The extracts of *Lonicera japonica* were analyzed by high-performance liquid chromatography (HPLC) (Hitachi L-7400; Tokyo, Japan) according to the method described by Lin et al. [15]. Twenty μL of the extract was loaded into a Thermo C18 capillary column (5 μm, 250 × 4.6 mm, Agilent, Waltham, MA, USA) and assayed in gradient elution mode during the chromatographic analysis. Elution was carried out using 0.1% acetic acid in water and methanol at a flow rate of 1.0 mL/min. Gradient elution was performed as follows: Methanol was set 30% for the first 5 min, then the methanol was increased to 50% between 5 and 10 min, and held at 100% for the last 5 min. The UV detector was set at a wavelength of 325 nm. Calibration curves were established using CGA standards, and samples were analyzed by comparing their retention times with those of the standards. The yield of CGA from *Lonicera japonica* was calculated according to the following formula Equation (2):

Extraction yield (mg/g) = mass of the compound in extraction solution (mg)/mass of dried material (g)(2)

### 3.5. Response Surface Methodology (RSM)

In this study, a 5-level-4-factor central composite rotatable design (CCRD), including 27 treatments, was employed. To avoid any unknown factor that may affect the experiment, the 27 runs were performed in random order. The independent variables of extraction temperature (30–70 °C), ethanol concentration (55–95%), L/S ratio (10:1–50:1 mL/g), and ultrasonic power (90–150 W) are presented in Table 1. Table 2 shows the independent factors, levels, and design matrix for the experiment. All reactions were carried out in duplicate. Regression analysis was performed for the experiment data by using Design-Expert (Version 8.0.6.1, Stat-Ease Inc., Minneapolis, MN, USA) software to fit the following second-order response model:

Y = β_0_+β_1_X_1_+β_2_X_2_+β_3_X_3_+β_4_X_4_+β_12_X_1_X_2_+β_13_X_1_X_3_+β_14_X_1_X_4_+β_23_X_2_X_3_+β_24_X_2_X_4_+β_34_X_3_X_4_

+β_11_X_1_^2^+β_22_X_2_^2^+β_33_X_3_^2^+β_44_X_4_^2^ (3)

where *Y* represents the response variable and β_0_ is the constant term; β_1_, β_2_, β_3_ and β_4_ are coefficients of the linear effects, β_11_, β_22_, β_33_ and β_44_ are coefficients of quadratic effects and β_12_, β_13_, β_14_, β_23_, β_24_ and β_34_ are coefficients of interaction effects for the four independent variables (*X*_1_ = temperature, *X*_2_ = ethanol concentration, *X*_3_ = L/S ratio, *X*_4_ = ultrasonic power).

### 3.6. Artificial Neural Network (ANN)

A multi-layer perceptron (MLP) based feed-forward ANN was applied for modeling ultrasonic-assisted extraction of CGA. A commercial ANN software, MATLAB version R2016a (MathWorks software, MathWorks Inc., Natick, MA, USA) was used in the study. The experimental data was constructed by the regression-based network approach. The network architecture consisted of an input layer with four neurons (temperature, ethanol concentration, L/S ratio, and ultrasonic power), an output layer with one neuron, which represented the extraction yield of CGA, and one hidden layer with ten neurons. A schematic diagram of the MLP architecture can be observed in Figure 2. BFGS quasi-Newton backpropagation (TRAINBFG) was an efficient training function because it has good performance in non-smooth optimizations and smaller networks [27]. Gradient descent method (LEARNGDM) as the adaptive learning function was used to minimize the mean squared error (MSE) between the network output and the actual error rate [28]. Mean squared normalized error performance function can appraise the network’s performance according to the MSE. The hyperbolic tangent sigmoid transfer function (TANSIG) and linear transfer function (PURELIN) were used to calculate a layer’s output from its net input [29]. All these functions were used to train the neural network and built the best ANN.

### 3.7. Comparison of Prediction Capability between ANN and RSM for CGA Extraction

Several statistical parameters, including the coefficient of determination (*R*^2^), root mean square error (RMSE) and absolute average deviation (AAD), were calculated for the comparison of estimation capabilities of RSM and ANN[15] as shown in Equations (4) to (6), respectively.

(4) $$R^{2}=1-\frac{\sum_{i=1}^{n}{{(Y}_{\mathrm{pre}}{-Y}_{\exp})}^{2}}{\sum_{i=1}^{n}{{(Y}_{m}{-Y}_{\exp})}^{2}}$$

(5) $$\mathrm{RMSE}=\;\sqrt{\frac{\sum_{i=1}^{n}{{(Y}_{\mathrm{pre}}{-Y}_{\exp})}^{2}}{n}}$$

(6) $$\mathrm{AAD}=\;\left[\frac{\sum_{i=1}^{n}\left(\left|Y_{\exp}{-Y}_{\mathrm{pre}}\right|{/Y}_{\mathrm{pre}}\right)}{n}\right]\text{×}100$$

where *Y*_pre_ is the predicted CGA extraction yield (by either RSM or ANN), *Y*_exp_ is the observed CGA extraction yield, *Y*_m_ is the average CGA extraction yield, and *n* is the number of experiments (n = 27 for CCRD experiments and n = 4 for external experiments).

## 4. Conclusions

The yield of CGA reached 43.13 mg/g under the optimal conditions, which were as follows: Temperature, 33.56 °C; ethanol concentration, 65.88%; L/S ratio, 46:1 mL/g; and ultrasound power, 150 W. Despite recent decade’s advances in the ultrasound-assisted extraction of CGA by applying response surface methodology, some complex nonlinear factors still lead to the significant lack of fit in the optimization process, especially the interaction between multiple factors. In this study, ANN modeling has successfully employed the ultrasound-assisted extraction of CGA. The constructed ANN shows high *R*^2^ values as 0.9898, whereas AAD and RMSE values had a smaller value as compared to those observed from the RSM. In conclusion, the application of ANN in ultrasound-assisted extraction of *Lonicera japonica* can obtain more CGA nutraceuticals, which has potential commercial applications.

## Figures and Tables

**Table 1:** Coding of experimental parameters and related levels.

Independent Variable	Unit	Symbols	Coded Values				
			−2	−1	0	+1	+2
Temperature	°C	X_1_	30	40	50	60	70
Ethanol concentration	%	X_2_	55	65	75	85	95
L/S ratio	mL/g	X_3_	10	20	30	40	50
Ultrasonic power	W	X_4_	90	105	120	135	150

**Table 2:** Central composite rotatable design (CCRD) and experimental data for 5-level-4-factor response surface analysis.

Run	Independent Variable ^a^				Chlorogenic Acid Extraction Yield (mg/g)				
	X_1_	X_2_	X_3_	X_4_	Experimental Data ^b^	RSM-Predicted	RSM Deviation	ANN-Predicted	ANN Deviation
1	40	65	20	105	20.75 ± 3.60	25.45	4.70	20.77	0.02
2	60	65	20	105	39.41 ± 1.31	34.99	4.42	39.37	0.04
3	40	85	20	105	18.70 ± 1.92	20.01	1.30	18.70	0.01
4	60	85	20	105	35.23 ± 1.74	32.99	2.24	35.24	0.00
5	40	65	40	105	38.02 ± 3.10	40.56	2.55	38.01	0.00
6	60	65	40	105	39.84 ± 2.06	43.18	3.33	41.49	1.65
7	40	85	40	105	34.12 ± 2.26	30.11	4.01	34.12	0.00
8	60	85	40	105	37.67 ± 2.00	36.16	1.51	37.66	0.01
9	40	65	20	135	30.65 ± 1.29	32.23	1.58	30.64	0.00
10	60	65	20	135	30.44 ± 4.18	34.82	4.39	30.84	0.40
11	40	85	20	135	29.68 ± 3.48	26.72	2.96	29.66	0.01
12	60	85	20	135	35.23 ± 2.62	32.75	2.48	35.24	0.01
13	40	65	40	135	40.74 ± 1.79	43.36	2.62	43.92	3.19
14	60	65	40	135	40.25 ± 2.78	39.02	1.23	40.62	0.37
15	40	85	40	135	28.33 ± 3.10	32.83	4.50	28.32	0.01
16	60	85	40	135	36.25 ± 0.74	31.94	4.32	35.93	0.32
17	30	75	30	120	37.59 ± 1.16	32.67	4.92	37.94	0.36
18	70	75	30	120	36.85 ± 1.17	41.31	4.47	36.84	0.01
19	50	55	30	120	38.32 ± 0.77	31.79	6.53	38.32	0.00
20	50	95	30	120	13.18 ± 2.34	19.26	6.08	13.18	0.00
21	50	75	10	120	26.31 ± 0.95	26.60	0.29	26.33	0.01
22	50	75	50	120	41.64 ± 2.47	40.90	0.74	41.65	0.01
23	50	75	30	90	36.35 ± 1.39	36.73	0.38	36.36	0.00
24	50	75	30	150	40.12 ± 1.57	39.29	0.83	38.58	1.54
25	50	75	30	120	39.78 ± 2.38	39.92	0.14	39.89	0.12
26	50	75	30	120	39.99 ± 2.33	39.92	0.07	39.89	0.10
27	50	75	30	120	39.98 ± 0.35	39.92	0.07	39.89	0.09

^a^ Independent variable X_1_: Temperature (°C), X_2_: Ethanol concentration (%), X_3_: Liquid/solid ratio (mL/g), X_4_: Ultrasonic power (W). ^b^ Mean of duplicate determinations.

**Table 3:** ANOVA for the experimental results of central-composite rotatable design (CCRD).

Source	Sum of Squares	DF	Mean Square	F Value	*p*-Value Prob > F
Model	1113.03	14	79.5	3.25	0.0238 *
X_1_	112.13	1	112.13	4.58	0.0535
X_2_	235.33	1	235.33	9.62	0.0092 *
X_3_	306.68	1	306.68	12.54	0.0041 *
X_4_	9.82	1	9.82	0.4	0.5382
X_1_X_2_	11.83	1	11.83	0.48	0.5
X_1_X_3_	48.02	1	48.02	1.96	0.1865
X_1_X_4_	48.3	1	48.3	1.97	0.1853
X_2_X_3_	25.19	1	25.19	1.03	0.3302
X_2_X_4_	4.889 × 10^−3^	1	4.889 × 10^−3^	1.998 × 10^−4^	0.989
X_3_X_4_	15.91	1	15.91	0.65	0.4356
X_1_^2^	11.41	1	11.41	0.47	0.5076
X_2_^2^	276.17	1	276.17	11.29	0.0057 *
X_3_^2^	50.68	1	50.68	2.07	0.1756
X_4_^2^	4.84	1	4.84	0.2	0.6645
Residual	293.55	12	24.46		
Lack of Fit	293.52	10	29.35	1983.46	0.0005 *
Pure Error	0.03	2	0.015		
Cor Total	1406.58	26			
Std. Dev.		4.95	R-Squared	0.7913	
Mean		34.27	Adj R-Squared	0.5478	
CV%		14.43			
PRESS		1690.75			

Independent variable X_1_: Temperature (°C), X_2_: Ethanol concentration (%), X_3_: Liquid/solid ratio (mL/g), X_4_: Ultrasonic power (W). * Significant at *p*-value less than 0.05.

**Table 4:** Validation experiments for the ultrasound-assisted extraction of CGA.

Run	Independent Variable ^a^				Chlorogenic Acid Extraction Yield (mg/g)				
	X_1_	X_2_	X_3_	X_4_	Experimental Data ^b^	RSM-Predicted	RSM Deviation	ANN-Predicted	ANN Deviation
1	60	65	30	120	39.65 ± 0.97	40.02	0.37	39.98	0.33
2	50	75	20	135	34.45 ± 2.17	35.96	1.51	33.29	1.16
3	60	75	30	135	37.70 ± 3.13	39.77	2.07	36.69	1.01
4	50	65	20	120	33.70 ± 2.55	33.08	0.62	33.82	0.12

^a^ Independent variable X_1_: Temperature (°C), X_2_: Ethanol concentration (%), X_3_: Liquid/solid ratio (mL/g), X_4_: Ultrasonic power (W). ^b^ Mean of duplicate determinations.

**Table 5:** Comparison of optimization and prediction capabilities of ANN and response surface methodology (RSM) for CGA extraction.

Parameters ^a^	RSM	ANN
*R* ^2^	0.7913	0.9898
RMSE	1.9050	0.7006
AAD	1.6541	0.4204

^a^ AAD: Absolute average deviation (%); RMSE: Root mean square error; *R*^2^: Coefficient of correlation determination.
